# Oxidative Stress Induces Bovine Endometrial Epithelial Cell Damage through Mitochondria-Dependent Pathways

**DOI:** 10.3390/ani12182444

**Published:** 2022-09-16

**Authors:** Pengjie Song, Chen Liu, Mingkun Sun, Jianguo Liu, Pengfei Lin, Aihua Wang, Yaping Jin

**Affiliations:** Key Laboratory of Animal Biotechnology of the Ministry of Agriculture, College of Veterinary Medicine, Northwest A&F University, Xianyang 712100, China

**Keywords:** dairy cow, oxidative stress, endometritis, PMN, BEECs damage

## Abstract

**Simple Summary:**

Polymorphonuclear neutrophil (PMN) count is the main diagnostic method of bovine endometritis. High neutrophil PMN counts in the endometrium of cows affected by endometritis suggest the involvement of oxidative stress among the causes of impaired fertility. The damage mechanism of oxidative stress on bovine endometrial epithelial cells (BEECs) is still unelucidated. The objective of this experiment was to investigate the relationship between oxidative stress and graded endometritis in dairy uteri and the molecular mechanism of oxidative stress injury to BEECs. Our research showed that there was an imbalance of antioxidant stress in dairy cow uterine with endometritis, oxidative stress damaged dairy cow endometrial epithelial cells through mitochondria-dependent pathways. These findings may provide new insight into the therapeutic target of bovine endometrial cell injury.

**Abstract:**

Bovine endometritis is a mucosal inflammation that is characterized by sustained polymorphonuclear neutrophil (PMN) infiltration. Elevated PMN counts in the uterine discharge of dairy cows affected by endometritis suggest that oxidative stress may be among the causes of impaired fertility due to the condition. Nevertheless, the effects of oxidative stress-mediated endometritis in dairy cows largely remain uninvestigated. Therefore, fresh uterine tissue and uterine discharge samples were collected to diagnose the severity of endometritis according to the numbers of inflammatory cells in the samples. Twenty-six fresh uteri were classified into healthy, mild, moderate, and severe endometritis groups based on hematoxylin and eosin stain characteristics and the percentage of PMNs in discharge. BEECs were treated with graded concentrations of H_2_O_2_ from 50 μM to 200 μM in vitro as a model to explore the mechanism of oxidative stress during bovine graded endometritis. The expressions of antioxidant stress kinases were detected by quantitative fluorescence PCR to verify the oxidative stress level in uteri with endometritis. Reactive oxygen species were detected by fluorescence microscope, and inflammation-related mRNA expression increased significantly after H_2_O_2_ stimulation. Moreover, mRNA expression levels of antioxidant oxidative stress-related enzymes (glutathione peroxidase, superoxide dismutase, and catalase) and mitochondrial membrane potential both decreased. Further investigation revealed that expression of the apoptosis regulator Bcl-2/Bax decreased, whereas expression of the mitochondrial apoptosis-related proteins cytochrome c and caspase-3 increased in response to oxidative stress. Our results indicate that an imbalance exists between oxidation and antioxidation during bovine endometritis. Moreover, apoptosis induced in vitro by oxidative stress was characterized by mitochondrial damage in BEECs.

## 1. Introduction

Dairy cow health, especially reproductive disease, has attracted increasing attention as the demand for milk and other dairy products has expanded [1]. Up to 40% of dairy cows develop postpartum uterine disease, which causes infertility by compromising the function of the endometrium [2]. High-quality milk requires a healthy uterus. Transition cows, in particular, face the challenge of negative energy balance (fatty liver, ketosis, subacute, acute ruminal acidosis) and perturbed immune function (metritis, mastitis) [3], given that all cows experience a reduced feed intake and body condition, infection, and inflammation of the uterus after calving [4]. Endometritis is an inflammation of the inner lining of the uterus and is one of the principal reproductive diseases that impact the dairy economy [5]. Recent surveys have revealed that endometritis has a prevalence of 20%, which ranges from 8% to 40%, in certain farms [6]. Bacterial infection is the main cause of postpartum endometritis in dairy cows. Bacteria, including *Escherichia coli*, *Arcanobacterium pyogenes*, *Fusobacterium necrophorum*, and *Staphylococcus aureus*, contaminate the uterus in >90% of dairy cattle in the first two weeks after parturition [7]. Diagnosis of endometritis is based on the presence of pus mixed with the vaginal mucus and the proportion of polymorphonuclear neutrophils (PMNs) among epithelial cells [8,9,10].

PMNs contribute to the first line of defence in the process of bacterial infection in the bovine uterus [11]. Neutrophils are recruited to inflammatory sites when inflammation occurs and then kill microbes by oxidant-dependent pathways [12]. In the activated state, PMNs produce and release superoxide anions and reactive oxygen species (ROS) to destroy the invading bacteria [13]. In cells, the initially produced superoxide radical is dismutated to hydrogen peroxide having a long half-life, which then diffuses and derives highly reactive ROS, hydroxyl radical [14]. However, an excessive free radical (OH^−^, O_2_^−^) load induces damage to structural and functional macromolecules, including lipids, protein, and RNA, but perhaps with the most severe repercussions on DNA [15]. The damaged macromolecules impact higher order structures such as compromising the insulation of organelles, e.g., endoplasmic reticulum, that maintain naturally high ROS. Pathological perturbations of ROS may result [16,17]. The inflammatory reaction will be more severe when invasion by pathogenic organisms or excessive free radicals produced by PMN cause irreversible damage to endometrial epithelial cells [18].

Inflammation is a complex systemic response. One of the most prominent features of the inflammatory response is the generation of a pro-oxidative environment due to the production of high fluxes of pro-oxidant species [19]. NADPH (nicotinamide adenine dinucleotide phosphate) oxidases (NOXs) are crucial enzymes that promote the production of ROS in neutrophils during inflammation [20]. Previous studies showed that NOX2 and mitochondria-derived ROS were required for the respiratory burst that occurs in activated leukocytes [21]. Enhanced ROS generation in mitochondria at the site of inflammation causes endothelial dysfunction and tissue injury [22]. ROS and superoxide anions have high reactivity with the bacterial cell membrane [23], nucleic acids, and proteins, thereby inducing microbial damage and death. However, these reactive molecules not only kill bacteria but also cause oxidative damage to BEECs [24]. High neutrophil counts in the endometrium of cows affected by endometritis suggest that oxidative stress may be among the causes of impaired fertility due to the condition [25]. High concentrations of ROS are a key indicator of many inflammatory diseases, and prolonged ROS production is a major factor that underpins chronic inflammation. In summary, oxidative stress is a crucial source of inflammation.

ROS emission accounts for approximately 2% of the total oxygen consumed by mitochondria under physiological conditions [26]. The balance between ROS generation and ROS scavenging is highly controlled, and oxidative stress is absent under normal conditions [27]. Mitochondria store the energy generated in the inner mitochondrial membrane as electrochemical potential energy during the processes of respiration and oxidation, thereby causing an asymmetric distribution of protons and other ions on different sides of the membrane, which forms the mitochondrial membrane potential (MMP) [28]. Superoxide anion is the main ROS that causes a decrease in the MMP [29]. A reduction in the MMP means that the capacity of mitochondria to transport ATP is decreased, which is a hallmark of cells during early apoptosis [30]. The accumulation of ROS and a decrease of the MMP indicate that mitochondria are damaged significantly [31]. Mitochondrial damage is an important feature of inflammation induced by oxidative stress. Severely damaged mitochondria lead to apoptosis [32]. The pathways of apoptosis are divided into the death receptor-mediated extrinsic, mitochondrial-mediated intrinsic, and endoplasmic reticulum stress-mediated pathways [33]. Mitochondrial-mediated apoptosis is characterized by mitochondrial outer membrane permeability, followed by cytochrome c release into the cytoplasm and activation of caspase [34]. The expression of pro-apoptotic proteins Bax and Bak is increased by apoptotic stimuli, following which these proteins bind to pro-survival Bcl-2 proteins to release Bax/Bak from inhibition [35]. Free Bax and Bak form oligomers, which lead to cytochrome c release from mitochondria to the cytoplasm, which activates the caspase cascade to induce apoptosis [36].

The present study aimed to investigate the relationship between oxidative stress and graded endometritis in dairy uteri and the molecular mechanism of oxidative stress injury to BEECs.

## 2. Materials and Methods

### 2.1. Tissue and Uterine Discharge Collection

All the uterine samples in our study are from Holstein-Friesian cows. To exclude the interference of other diseases, cows with infertility were selected, and dairy cows with mastitis, hoof disease, and other diseases were excluded. Fresh cow uteri were collected from slaughterhouses and transported to the laboratory on ice within two hours. The uterine cavity was exposed completely and was scraped for uterine discharge (UD). A 4 × 4 mm tissue section was obtained using biopsy forceps. Collected tissue was placed into a saline solution (0.9%). The uteri tissue samples were divided into two parts: one part was immersed immediately in liquid nitrogen for total protein and RNA extraction, and the second section was placed into paraformaldehyde (4%) for histopathology using routine haematoxylin and eosin (HE) staining.

### 2.2. Cytological Smear Preparation and Cytological Assessment

Duplicate cytology smears were prepared immediately after the UD samples were collected. Slides for cytologic examination were prepared by rolling a disposable inoculation ring with the smear onto a clean glass microscope slide. The slides were air-dried and stained using the Diff-Quick staining protocol (Solarbio, Beijing, China). The slides with smear are naturally dry and then fixed with Diff-Quik Fixative for 20 s. Then, Diff-Quik I staining for 5–10 s and Diff-Quik II staining for 10–20 s was completed. After rinsing, the slides were observed under a microscope. The cytological assessment was performed by counting a minimum of 100 cells to determine the percentage of PMNs. The PMN percentages were evaluated microscopically (magnification of 400×; Ni-U, Nikon, Tokyo, Japan) by a single experienced observer who was blinded to the slides’ origin. At least 100 PMN and epithelial cells were counted for each microscope field and used to calculate the PMN proportion in the sample [(PMN cells)/(PMN + epithelial cells)]. At least three fields of view were analyzed for each sample. Briefly, smears were evaluated microscopically, and the proportions of PMNs, lymphocytes and epithelial cells were recorded.

### 2.3. Evaluation and Diagnosis of Dairy Endometritis

Endometritis in dairy cows is a controversial issue among practitioners due to the lack of a diagnostic gold standard [37]. In most cases, the definitive diagnosis of endometritis is made based on histological examination of endometrial tissue and the percentage of PMN in vaginal discharge. The threshold value for PMNs as diagnostic for subclinical endometritis depends on the time postpartum and varies from 5 to 18% [38]. It has also been shown that a general threshold of 5% PMN is permissible for all cows between 21 and 62 days postpartum. Briefly, PMN and lymphocytes in the present study were counted in histological sections of endometrial tissue specimens. Similarly, the proportion of PMNs and lymphocytes to all cells were counted in UD. We deemed that severe endometritis (Se) was present if the proportion of PMNs was >25% [38,39,40] in UD and was accompanied by exfoliation or necrosis of endometrial epithelial cells. In contrast, we assessed that moderate endometritis (Moe) was present if the proportion of PMNs was 18–25% [41,42,43], endometrial epithelial cells were flattened, and the proportion of mononuclear cells was 5–10%. Mild endometritis (Mie) was defined if the proportion of PMNs was 2–5% [44,45,46] and mononuclear cells were 3–5% in lamina propria. Endometrial epithelial cells were columnar when the proportion of PMNs was <3% [47,48,49] (Table 1).

### 2.4. Cell Culture and Treatment

Fresh cow uteri were collected from slaughterhouses and transported to the laboratory on ice within two hours. BEECs were isolated immediately from healthy cornua uteri and were cultured in DMEM supplemented with penicillin (100 mg/mL) and streptomycin (100 U/mL) with 10% FBS at 37 °C in a humidified atmosphere with 5% CO_2_. After expanded culture, the BEECs were stored in liquid nitrogen for subsequent experiments. BEECs were recovered and treated with 0, 50, 100, and 200 μM H_2_O_2_ when required. BEECs were seeded on the 6-wells plates at a density of 2 × 10^5^.

### 2.5. Total RNA Extraction and Quantitative Reverse Transcription PCR

Uterine tissue samples were thawed at low temperatures. Uteri samples and liquid nitrogen were added to mortar; the samples were ground into powder at low-temperature RNA, and protein was extracted. Total RNA from BEECs and endometrial tissues was isolated using RNAiso Plus (Takara, Maebashi, Japan), according to protocols from the manufacturer. Extracted RNA was quantified, and 1 μg of RNA was added to a genomic DNA elimination reaction for reverse transcription into a cDNA template with gDNA Eraser (Takara). Quantitative PCR was performed with a Bio-Rad CFX96 system using the SYBR Green Plus Reagent Kit (Takara). The reaction conditions were as follows: 95 °C for 2 min, followed by 40 cycles at 95 °C for 10 s and 60 °C for 30 s. The mRNA expression levels were measured relative to the mRNA of the β-actin reference gene using the 2^−^^ΔΔCt^ method. The primer sequences for each gene are reported in Table 2.

### 2.6. Detection of Intracellular ROS

Intracellular ROS was detected using 2,′ 7′-dichlorofluorescein diacetate (DCFH-DA) according to the manufacturer’s protocol (Beyotime, Shanghai, China). BEECs were seeded on coverslips in 12-well plates, and the cells were attached completely within 12 h. The culture was discarded at 70–80% confluency, and adhered BEECs were washed three times with PBS. BEECs were incubated with a serum-free medium containing 10 μmol/L DCFH-DA and then treated with various concentrations of H_2_O_2_ (0, 50, 100, or 200 μM) at 37 °C for five hours. Rosup was used as a positive control for inducing oxidative stress. The cells were observed by fluorescence microscopy (Nikon, Tokyo, Japan), and the fluorescence intensity was evaluated with ImageJ 1.47 v software.

### 2.7. Detection of Mitochondrial Membrane Potential

The decrease of MMP is characteristic of the early stage of apoptosis. 5,5′,6,6′-Tetrachloro-1,1′,3,3′- tetraethyl-imidacarbocyanine iodide (JC-1) is an ideal fluorescent probe that is used widely to detect MMP. At higher mitochondrial membrane potentials, JC-1 accumulates in the matrix of mitochondria to form polymers (J-aggregates) that produce red fluorescence. When the mitochondrial membrane potential is low, JC-1 could not accumulate in the matrix of mitochondria that exists as a monomer and emits green fluorescence exposed to blue light. MMP was detected with the JC-1 kit (Beyotime); the JC-1 solution was diluted 200 times. BEECs were stimulated with various concentrations of H_2_O_2_ (0, 50, 100, or 200 μM) for five hours, followed by the addition of a JC-1 working solution (0.6 mL). After incubation for 20 min at 37 °C, the BEECs were washed with phosphate-buffered saline three times. The fluorescence intensity was measured by confocal laser scanning microscopy.

### 2.8. Transmission Electron Microscopy

BEECs were fixed using 2.5% glutaraldehyde at room temperature, washed by PBS, fixed by osmic acid, washed by PBS, and dehydrated by a graded ethanol series. After embedment in LR-White, the sample was cut into ultrathin sections. The ultrathin sections were stained using 3% uranyl acetate–lead citrate cream and photographed using an HT7800 transmission electron microscope (Hitachi, Tokyo, Japan).

### 2.9. Protein Extraction and Western Blotting

Total protein was extracted from cells and uterine tissue with a protein extraction kit (KeyGEN, Changchun, China) according to protocols from the supplier. Protein concentration was determined using a bicinchoninic acid assay (KeyGEN). Equal concentrations of total protein were separated by SDS-PAGE (12%). Proteins were transferred onto polyvinylidene difluoride membranes and were blocked for two hours with TBST (50 mmol/L Tris, pH 7.6, 150 mmol/L NaCl, and 0.1% Tween 20) containing 5% BSA. The membranes were incubated with primary antibodies diluted in TBST overnight at 4 °C. Antibodies against cytochrome C (ab133504), caspase-3 (ab184787), BAX, (ab32503), and Bcl-2 (ab182858) were from Abcam (Shanghai, China). After washing three times in TBST, the membranes were incubated with horseradish peroxidase-conjugated secondary antibodies for two hours at room temperature and then washed three times for 10 min. Protein bands were visualized by exposure to an enhanced chemiluminescence detection system imager (Tanon Biotech, Shanghai, China) with an enhanced chemiluminescence solution (DiNING, Beijing, China). The relative intensity of each band was assessed by Image J 1.47 v software.

### 2.10. Statistical Analysis

Statistical analysis was performed using SPSS Statistics 25 (Chicago, IL, USA). In our study, each experiment was repeated three times; a one-way ANOVA was used, and the results were compared between groups or with the control for multiple comparisons with Bonferroni correction. Differences between means were determined using Duncan’smultiple comparisons. All data are presented as means ± SD. A *p*-value < 0.05 was considered statistically significant, and a *p*-value < 0.01 was considered highly significant.

## 3. Results

### 3.1. Examination of Bovine Uteri and Uterine Discharge

**Figure 1:**
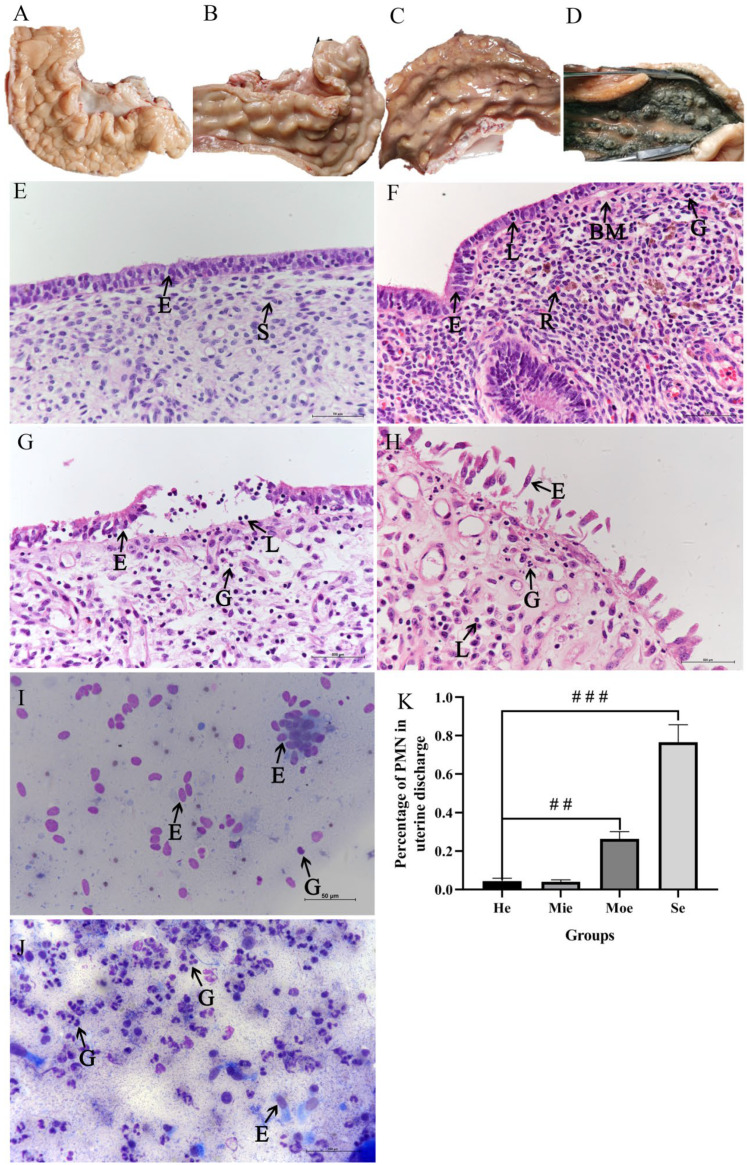
Histopathologic and cytological characterization of the bovine uterus. (**A**–**D**) Healthy dairy cow uterus (He), dairy cow uterus with mild endometritis (Mie), dairy cow uterus with moderate endometritis (Moe), and dairy cow uterus with severe endometritis (Se), respectively. (**E**–**H**) Corresponding histopathological characterization of samples in panels (**A**–**D**). (**I**) Representative cytology by cytobrush image of healthy dairy cow uterus. (**J**) Representative cytology by cytobrush image of the uterus with endometritis. (**K**) Percentage of PMNs of UD in He, Mie, Moe, and Se groups. Different fields were randomly selected, 100 cells were counted, and the percentage of PMN was calculated. E indicates a luminal epithelial cell, G denotes a granulocyte, L is a lymphocyte, S indicates a stroma cell, and R is a red cell, BM is a basement membrane. Images were magnified 400×. One-way ANOVA analysis was used to compare to the control group. ## *p* < 0.01, ### *p* < 0.001.

Uteri were classified according to different levels of inflammation to investigate the relationship between graded endometritis and oxidative stress in the bovine endometrium. Uteri were divided into four groups based on the results of HE staining and cytological assessment. Three uteri were graded as having Se (Figure 1D). The PMN counts of UD in this group were >50%, most luminal epithelial cells were desquamated, epithelial necrosis and nuclear condensation were evident, and great numbers of PMNs and lymphocytes were infiltrated in the lamina propria (Figure 1D,H). Five uteri were graded with Moe with significant lymphocyte infiltration, relatively few luminal epithelium cells desquamated, and few PMNs in UD (Figure 1C,G). Six uteri were graded as displaying Mie, with few lymphocytes in the epithelial layer, scanty presence of red blood cells in lamina propria, and hardly any PMNs in UD (Figure 1B,F). Twelve uteri were graded as normal (He) with the scanty presence of inflammatory cells (Figure 1A,E). Few granulocytes are present in UD without endometritis (Figure 1I), whereas more granulocytes occur in UD with endometritis (Figure 1J).

### 3.2. Glutathione Peroxidase, Superoxide Dismutase, Catalase Show Reduced Expression and IL-8, IL-10 Show Increased Expression in Bovine Uteri with Inflammation

**Figure 2:**
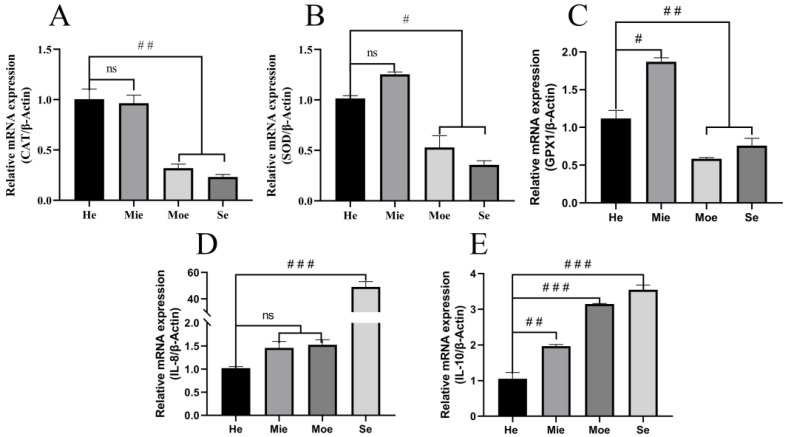
The mRNA expression levels of antioxidant stress related enzymes CAT (**A**), SOD (**B**), GPx (**C**) and inflammation-related IL-8 (**D**), IL-10 (**E**) in uterine tissue were assessed by RT-qPCR. The experiment was repeated three times. Expression data were normalized to that of β-actin. One-way ANOVA analysis was used to compare to the control group. Significance differences are marked as # *p* < 0.05, ## *p* < 0.01, ### *p* < 0.001, ns no significance.

Real-time PCR was performed to examine differences in the expression of anti-oxidative stress-related enzymes in uteri with endometritis compared to healthy uteri. The results showed that the expression of glutathione peroxidase (GPx) in the Moe and Se groups was reduced significantly (*p* < 0.01) compared with the He group (Figure 2C). Superoxide dismutase (SOD) expression also was less (*p* < 0.05) in the endometrium in the Moe and Se groups compared with the He group (Figure 2B). Similarly, catalase (CAT) expression in the endometrium was reduced significantly (*p* < 0.01) in the Moe and Se group compared with normal (Figure 2A). In contrast, expression of SOD and CAT was not significantly different in the Mie group compared with the He group, but GPx gene expression was increased significantly (*p* < 0.05) (Figure 2). The expression of IL-8 in the Se group was increased (*p* < 0.001), but there was no significant difference in the Mie group and Moe group compared with the He group (Figure 2D). IL-8 expression in the Moe group and Se group was increased significantly (*p* < 0.001) compared with the normal (Figure 2E).

### 3.3. H_2_O_2_ Induces Increased Inflammatory Cytokines and Decreased Antioxidant Enzymes in BEECs

**Figure 3:**
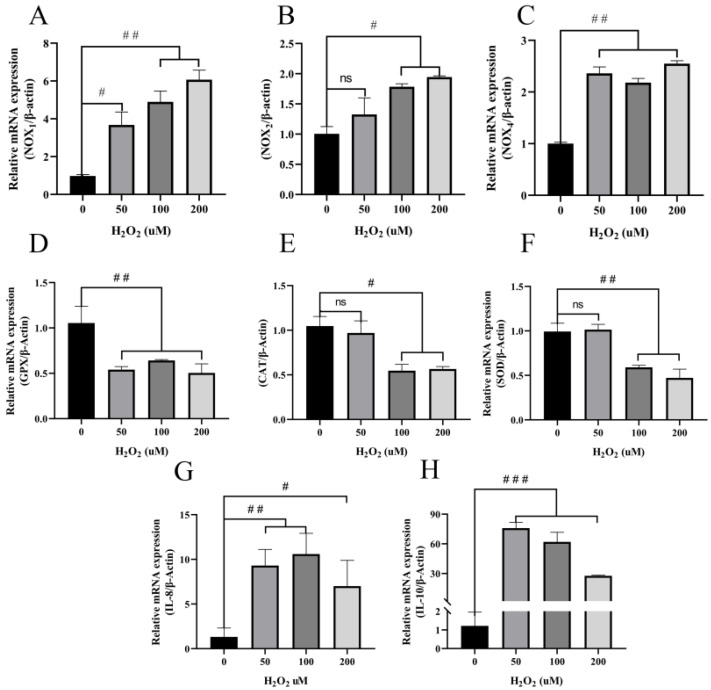
Oxidative stress-related factors and inflammatory cytokines in BEECs treated with different concentrations of H_2_O_2_ for five hours were detected. (**A**–**C**) Expression of ROS-generating oxidases NOX1, NOX2, and NOX4, respectively. (**D**–**F**) Expression of antioxidant stress-related factors GPx, CAT, and SOD, respectively. (**G**,**H**) Expression of inflammatory cytokines IL-8 and IL-10, respectively. The experiment was repeated at least three times. All data are means ± S.E. One-way ANOVA analysis was used to compare to the control group. # *p* < 0.05, ## *p* < 0.01, ### *p* < 0.001, ns no significance.

An in vitro oxidative stress model targeted to bovine endometritis was established, and various concentrations of H_2_O_2_ (50, 100, and 200 μM) were used to induce oxidative stress in BEECs [50,51]. The expression of the genes for inflammation-related factors IL-8 and IL-10 increased in BEECs (*p* < 0.01) following stimulation with 50 or 100 μM H_2_O_2_ for five hours compared with the untreated cells. The expression of IL-8 and IL-10 also increased significantly in the 200 μM group but less than with 50 or 100 μM H_2_O_2_ (Figure 3G,H). Interestingly, IL-8 increased without dose-dependence after H_2_O_2_ stimulation (Figure 3G). Antioxidant enzymes are not only the main contributors to the elimination of oxygen free radicals but also are markers of antioxidant stress. The expression of GPx decreased significantly (*p* < 0.01) after incubation of BEECs with H_2_O_2_ compared with the control group (Figure 3D). Additionally, 100 and 200 μM H_2_O_2_ significantly inhibited the expression of SOD (*p* < 0.01) and CAT (*p* < 0.05), although 50 μM H_2_O_2_ had no significant effect on the expression of these enzymes (Figure 3E,F). NOXs are one of the major sources of cellular ROS, high levels of which are the principal cause of oxidative stress. The expression of the genes for NOX1 and NOX4 was increased significantly after incubation with H_2_O_2_ compared with the untreated control (Figure 3A,C). In contrast, we also observed that the expression of NOX2 did not alter significantly after incubation with 50 μM H_2_O_2_, but that expression increased significantly at concentrations of 100 and 200 μM H_2_O_2_ (Figure 3B). Taken together, these data suggest that H_2_O_2_ induces higher expression of inflammatory and oxidative stress-related factors but lower expression of antioxidant enzymes in BEECs.

### 3.4. H_2_O_2_ Increases ROS and a Decrease of the MMP to Damage Mitochondria in BEECs

**Figure 4:**
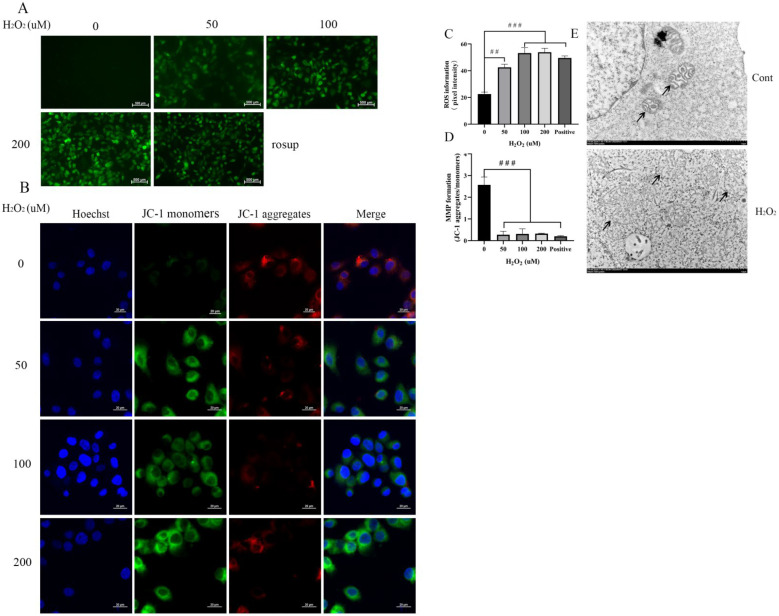
H_2_O_2_-induced intracellular ROS increase and MMP decrease in BEECs. (**A**) BEECs were exposed to the indicated concentrations of H_2_O_2_ for five hours. Rosup was a positive control. ROS levels were detected by DCFH-DA fluorescence (green). (**B**) MMP (mitochondrial membrane potential) was detected with the JC-1 kit. Cellular mitochondria with normal MMP emitted red fluorescence (J-aggregate), while those with abnormal MMP showed green fluorescence (J-monomer). (**C**) Quantification of intracellular ROS levels relative to the untreated group. (**D**) Quantitative analysis of the MMP. The MMP was calculated using Image-J as red/green fluorescence. (**E**) After being exposed to the indicated concentrations of H_2_O_2_ for five hours, the mitochondria of BEECs were analyzed by transmission electron microscopy. The typical mitochondrial structures are clearly visualized: black arrowheads depict the mitochondria. The data are representative of three independent experiments. The optical density was calculated for each sample with ImageJ 1.47v software. The experiment was repeated in triplicate. One-way ANOVA analysis was used compared to the control group. ## *p* < 0.01, ### *p* < 0.00.

ROS oxidizes non-fluorescent DCFH-DA intracellularly to produce green, fluorescent DCF. The strength of the fluorescence correlates with higher ROS levels. Green fluorescence of H_2_O_2_-treated cells increased significantly compared with the untreated group, although fluorescence intensity was not significantly different between the H_2_O_2_-treated groups (Figure 4A). These results suggest that 50, 100, and 200 μM H_2_O_2_ increase intracellular ROS levels to similar values in BEECs (Figure 4C). We also examined the MMP to ascertain whether exposure of BEECs to H_2_O_2_ affects membrane integrity. Data obtained from JC-1 staining showed that the MMP decreased significantly after H_2_O_2_ stimulation compared with the untreated group (Figure 4B), although there were no significant differences among the H_2_O_2_-treated groups. Meanwhile, mitochondrial morphology was observed by transmission electron microscope (Figure 4E). After treatment with H_2_O_2_, for five hours the mitochondria showed swelling and vacuolization of mitochondria, clearing of the mitochondrial matrix, and breakage of mitochondrial cristae. These results suggest that mitochondria are damaged after H_2_O_2_ exposure. In summary, the increase in ROS and the decrease in MMP indicate that mitochondria are damaged after treatment of BEECs with H_2_O_2_.

### 3.5. Pro-Apoptosis of Mitochondria-Dependent Proteins Increases after Treatment with H_2_O_2_

**Figure 5:**
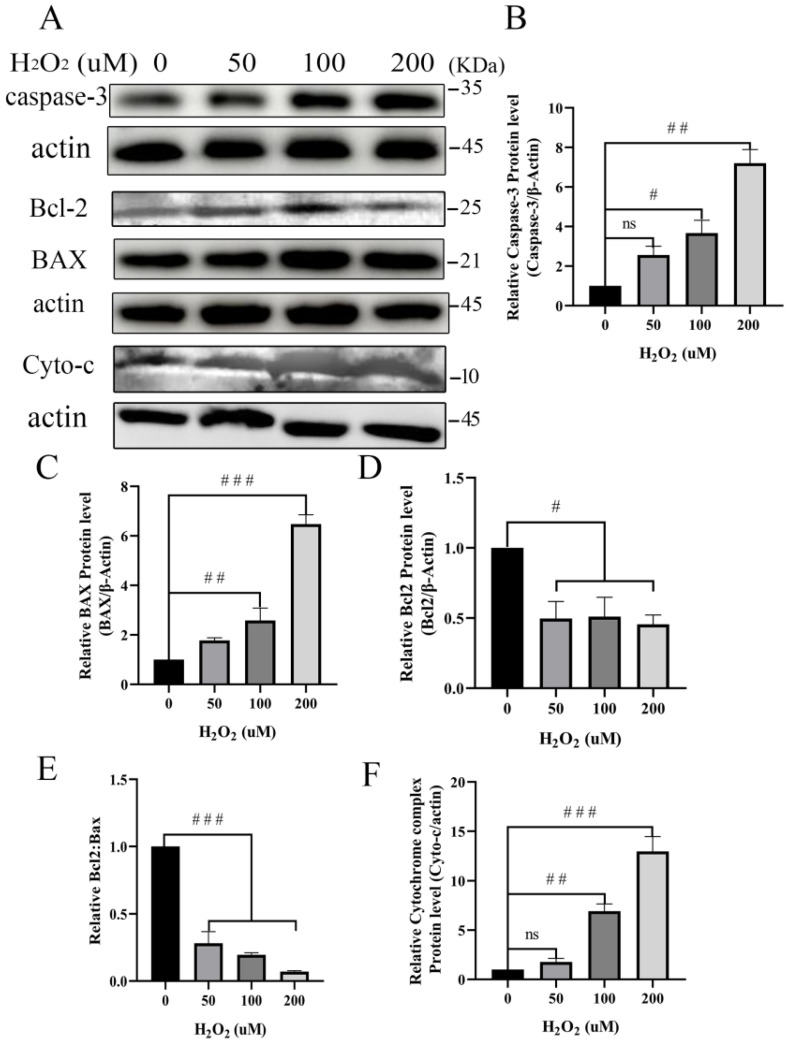
BEECs were treated with H_2_O_2_ at different concentrations for five hours. Cells were prepared for Western blotting with antibodies against caspase-3, Bcl-2, BAX, and cyto-c. Expression relative to the β-actin reference was quantified using gray-scale analysis by ImageJ 1.47v software. The data are representative of three independent experiments. One-way ANOVA analysis was used to compared to the control group. # *p* < 0.05, ## *p* < 0.01, ### *p* < 0.001. (**A**) The protein expression of caspase-3, Bcl2, BAX, Cyto-c; (**B**) Expression level of caspase-3 relative to β-Actin; (**C**) Expression level of BAX relative toβ-Actin; (**D**) Expression level of Bcl-2 relative to β-Actin; (**E**) Expression level of Bcl2:BAX; (**F**) Expression level of Cyto-c relative to β-Actin. Original Western Blot could be found as Supplementary Material.

Mitochondria may be severely damaged due to ROS that is released during inflammation. We detected mitochondrial-dependent apoptosis proteins in BEECs after treatment with H_2_O_2_ to examine this hypothesis further. The expression of mitochondria-dependent apoptosis proteins cytochrome C, caspase-3, and BAX increased in BEECs with increasing H_2_O_2_ concentrations compared with the untreated control (Figure 5). Additionally, we monitored the expression of the Bcl-2 protein that regulates apoptosis. Exposure of BEECs to H_2_O_2_ resulted in reduced expression of Bcl-2 (Figure 5).

## 4. Discussion

The uterus in a healthy state is a sterile environment. Postpartum cow uterine infection is a major cause of endometritis. An unhealthy uterus is detrimental to embryo implantation. Therefore, dairy cow uterine infection is a significant cause of economic loss and animal distress [52]. The grading of endometritis is conducive to understanding the pathogenesis of bovine endometritis to provide strategies for the treatment of the condition. Hence, in the present study, we aimed to investigate the relationship between oxidative stress and graded endometritis in dairy uteri and the molecular mechanism of oxidative stress injury to BEECs.

Dairy cow endometritis is quantifiable by the proportion of neutrophils in cytology samples which has led to the use of the term cytological endometritis as a more descriptive diagnosis [10]. Cytological assessment has been recommended as a reliable method for determining the percentage of polymorphonuclear leukocytes at the endometrial surface [53]. The diagnosis of endometritis in dairy cows is a controversial topic in bovine health due to the lack of a diagnostic gold standard [54,55], and no diagnostic test is considered 100% accurate [56]. Endometritis is defined histologically as the presence of inflammatory cells in the uterine endometrium with disruption, or not, of the epithelial layer [57]. Histopathology is considered the best way to diagnose endometrial alterations [58], mainly because this approach allows direct visualization of both acute and chronic alterations in the epithelium and stratum compactum of the endometrium (Figure 1). In this study, mean PMN counts based on cytological smear and histopathological examination were significantly different between healthy and endometritis groups (Figure 1F). However, PMN count alone is insufficient to indicate the inflammation level. Uterine biopsy has been reported to be a reliable technique for assessing uterine function and health, but the procedure is detrimental to further fertility [59] and failure of embryo implantation [60]. Consequently, fresh uteri from slaughterhouses were studied here to grade endometritis according to PMN count and HE staining (Figure 1). The high expression of the anti-inflammation factor IL-10 antagonizes other inflammatory factors to inhibit inflammation response. IL-8 induces fever, participates in pathological inflammatory damage, and promotes the release of inflammatory mediators. High expression of IL-8 and IL-10 in endometritis groups proves that it is consistent with the pathological examination and cytological examination.

ROS are among the main cellular electrophiles involved in a delicate and easily corruptible balance between biological benefit and damage [61]. ROS are of three major types, namely hydrogen peroxide, OH^−^, and O_2_^−^, and affect cells mainly by DNA damage and reducing MMP. It has been reported that oxidative damage induced by ROS and subsequent cell death are associated with several human diseases, such as diabetes [62,63]. Mitochondria are equipped with SOD which is one of the most efficient ROS scavengers. SOD is the only factor that converts superoxide into hydrogen peroxide [19]. In addition, GPx and CAT convert hydrogen peroxide into water and thereby play important roles in preventing the formation of hydroxyl radicals. Wu et al. [64] found that salvianolic acid C enhanced the expression of SOD, GPx, and the antioxidant glutathione to mitigate mitochondrial oxidative stress and the inflammatory response. We tested for SOD, GPx, and CAT to investigate the antioxidant levels in uterine tissue with endometritis. The expression of these enzymes was decreased significantly in the Moe and Se groups compared with the He group, and the levels of IL-8 and IL-10 were significantly increased in the Moe and Se groups. Thus, these data indicate that there is a disequilibrium of oxidative stress-related enzymes in bovine uteri with endometritis. Exposure to H_2_O_2_ is a widely used procedure to induce oxidative damage/stress in cellular models [65]. BEECs were exposed to graded concentrations of H_2_O_2_ to simulate the cellular environment during endometritis. Previous studies indicated that ROS were generated primarily by NOX2 and activated inflammation in microglia [66]. Here, we observed that high expression of NOX1, NOX2, and NOX4 promoted intracellular ROS accumulation and that MMP simultaneously was decreased. Based on a previous report [66,67], NOXs promote intracellular ROS accumulation and exacerbate the inflammatory response in certain types of mammalian cells. The function of NOXs in regulating redox processes and inflammation is a complicated process. The findings here provide evidence that NOX1, NOX2, and NOX4 promote ROS generation to express IL-8 and IL-10 in BEECs (Figure 3). Thus, the results demonstrate that H_2_O_2_ induces inflammation and oxidative stress in BEECs.

Mitochondria are the energy centers of cells and are essential for cellular survival. MMP is a key indicator of cell health and injury due to the important role that it plays in adenosine 5’-triphosphate synthesis [68]. Previous studies have reported that monitoring of MMP is a good indicator for the assessment of cell status and diagnosis of diseases [69]. As shown in Figure 4B,D, treatment of BEECs with H_2_O_2_ significantly decreased MMP compared with the control group. Mitochondrial damage causes the decrease of MMP, mitochondrial swelling, and ridge disappearance [70,71]. Electron microscope examination here showed that after treatment of BBECs with H_2_O_2_, mitochondria were damaged with mitochondrial swelling and ridge disappearance (Figure 4E).

At higher ROS levels, longer mitochondrial permeability transition pore openings may release a ROS burst leading to the destruction of mitochondria and, if propagated from mitochondrion to mitochondrion, of the cell itself [27]. Neitemeier et al., found that mitochondrial damage induced neuronal apoptosis by ferroptosis [72]. Moreover, persistent oxidative stimulation not only attenuates the effects of cellular antioxidative systems but also damages mitochondria resulting in high expression of proapoptotic proteins [73]. Consistent with a previous report [74,75], we found that the expression of apoptosis-related protein Bax increased significantly after treatment of BEECs with H_2_O_2_ (Figure 5A). Bcl-2 family members, including Bcl-2, Bax, Bcl-w, and Bcl-xL, are crucial integrators of signals for cell survival and death [76]. The ratio of Bcl-2/BAX appears to determine the survival or death of cells following an apoptotic stimulus [77]. Previous studies showed that a reduction in the Bcl-2/BAX ratio with a simultaneous decrease in MMP indicated that mitochondria-damage mediated pathways induced apoptosis [78,79]. The shift in Bax/Bcl-2 ratio in favor of apoptotic signal, a reduction in the Bcl-2/BAX ratio increased levels of cytochrome c release and the expression of proapoptotic protein caspase-3 [80]. According to our data, the ratio of Bcl-2/BAX (Figure 5) decreased significantly with the decrease in MMP and increased ROS in BEECs undergoing oxidative stress (Figure 4). Our findings are consistent with previous reports in which the aggravation of oxidative stress during inflammation caused increased expression of caspase-3 and cytochrome c (Figure 5). It has been demonstrated that an increase in the release of cytochrome c from the mitochondria into the cytosol is regulated by Bcl-2 family proteins [81]. Therefore, the regulatory interplay between the Bcl-2 family and cytochrome c is essential for the control of apoptosis. In other words, high levels of ROS and decreased MMP under oxidative stress conditions damage the mitochondria by a reduction in the Bcl-2/BAX ratio and facilitate the enhanced expression of cytochrome c and caspase-3. Taken together, these data provide evidence that damage caused by oxidative stress induces BEECs to express mitochondria-dependent pro-apoptosis proteins.

## 5. Conclusions

In conclusion, our findings reveal that oxidative stress occurs in the bovine uterus with endometritis. Oxidative stress is correlated positively with the severity of endometritis, and H_2_O_2_-induced oxidative stress promotes apoptosis in a mitochondrial damage-dependent pathway during inflammation in BEECs.

## Figures and Tables

**Table 1:** Histopathologic and cytological scoring criteria.

Histopathologic Feature of Epithelium	Mononuclear in Lamina Propria (%)	Cytological PMN% in Mucus	Description
Columnar	Mononuclear < 3%	PMN < 2%	Normal
Cuboidal	3–4% < Mononuclear < 5%	5% < PMN <18%	Mild
Flattened	5% < Mononuclear < 10%	18% < PMN < 25%	Moderate
Necrosis and loss	10% < Mononuclear	25% < PMN	Severe

**Table 2:** Primer pairs used for q-PCR.

Gene Name	ID	Sequence	Size (bp)
CAT	NM_001035386.2	F: AGAGGAAACGCCTGTGTGAG R: ATGCGGGAGCCATATTCAGG	115
SOD	NM_174615.2	F: CTCTACTTGGTTGGGGCGTC R: TCGAAGTGGATGGTGCCTTG	122
GPx	NM_174076.3	F: AACGTAGCATCGCTCTGAGG R: GATGCCCAAACTGGTTGCAG	121
NOX1	NM_001191340.1	F: TGTCTTTCCTGAGAGGCACC R: TTTGTGGAAGGCGAGGTTGT	80
NOX2	NM_174035.4	F: CAAGATGGAGGTGGGCCAAT R: GAGGTCAGGGTGAAAGGGTG	81
NOX4	NM_001304775.1	F: TCTGGACCTTTGTGCCT R: GACGGATGACTTGTGACTG	95
IL-8	NM_173925.2	F: CATTCCACACCTTTCCACCC R: AGGCAGACCTCGTTTCCATT	116
IL-10	NM_174088.1	F: CACAGGCTGAGAACCACG R: AGGGCAGAAAGCGATGA	108

## Data Availability

The raw data supporting the conclusions of this article will be made available by the authors, without undue reservation. Full data available from the first author.

## Supplementary Materials

The following are available online at https://www.mdpi.com/article/10.3390/ani12182444/s1, Figure S1: original Western Blot figures.
