# Supplementary figures and images for: Oxidative Stress Induces Bovine Endometrial Epithelial Cell Damage through Mitochondria-Dependent Pathways
Source: Animals (Basel). 2022 Sep 16;12(18):2444. doi: 10.3390/ani12182444 (PMC9495185; doi:10.3390/ani12182444)

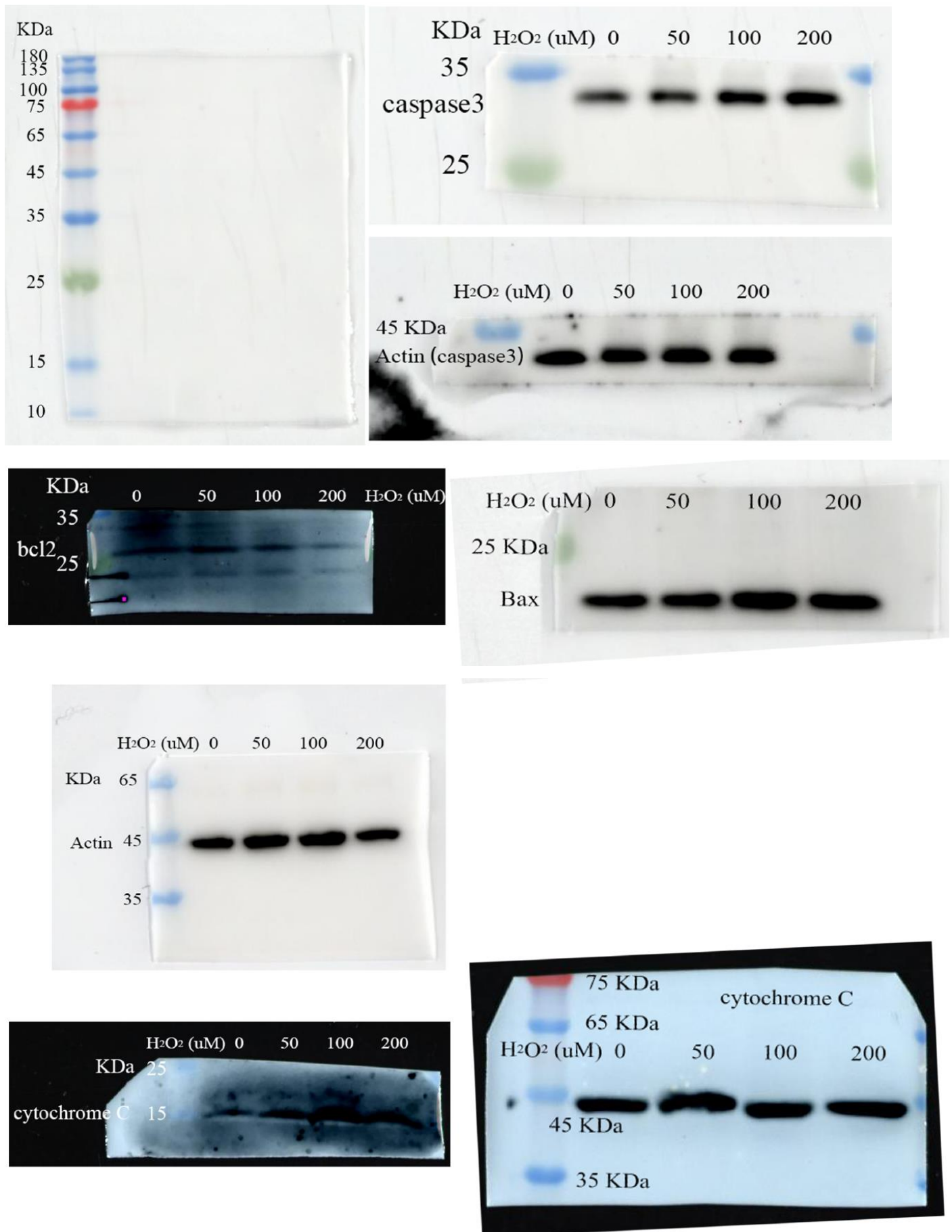

Figure S1: original Western Blot figures.

Supplement: Supplementary file 1 [file animals-12-02444-s001.zip › animals-1873070-supplementary.pdf]
